# Re-Structuring of Marine Communities Exposed to Environmental Change: A Global Study on the Interactive Effects of Species and Functional Richness

**DOI:** 10.1371/journal.pone.0019514

**Published:** 2011-05-18

**Authors:** Martin Wahl, Heike Link, Nicolaos Alexandridis, Jeremy C. Thomason, Mauricio Cifuentes, Mark J. Costello, Bernardo A. P. da Gama, Kristina Hillock, Alistair J. Hobday, Manfred J. Kaufmann, Stefanie Keller, Patrik Kraufvelin, Ina Krüger, Lars Lauterbach, Bruno L. Antunes, Markus Molis, Masahiro Nakaoka, Julia Nyström, Zulkamal bin Radzi, Björn Stockhausen, Martin Thiel, Thomas Vance, Annika Weseloh, Mark Whittle, Lisa Wiesmann, Laura Wunderer, Takehisa Yamakita, Mark Lenz

**Affiliations:** 1 Department of Benthic Ecology, IFM-GEOMAR, Kiel, Germany; 2 Institut des Sciences de la Mer de Rimouski, Université du Québec à Rimouski, Rimouski, Québec, Canada; 3 Marine Ecological Services, Paris, France; 4 School of Biological Sciences, Victoria University of Wellington, Wellington, New Zealand; 5 Leigh Marine Laboratory, University of Auckland, Leigh, New Zealand; 6 Department of Marine Biology, Universidade Federal Fluminense, Niteroi, Rio de Janeiro, Brazil; 7 Department of Conservation, Waikato Conservancy, Hamilton, New Zealand; 8 CSIRO Marine and Atmospheric Research, and School of Zoology, University of Tasmania, Hobart, Tasmania, Australia; 9 Centre of Life Sciences, University of Madeira, Funchal, Portugal and CIMAR/CIIMAR – Centre of Marine and Environmental Research, Porto, Portugal; 10 German Centre for Marine Biodiversity Research, Senckenberg am Meer, Wilhelmshaven, Germany; 11 ARONIA Coastal Zone Research Team, Åbo Akademi University and Novia University of Applied Sciences, Ekenäs, Finland; 12 Leiden, The Netherlands; 13 Department of Mikrobiology, Humboldt University, Berlin, Germany; 14 Biologische Anstalt Helgoland, AWI Marine Station, Helgoland, Germany; 15 Akkeshi Marine Station, Field Science Center for Northern Biosphere, Hokkaido University, Akkeshi, Hokkaido, Japan; 16 Raseborgsvägen 9, Ekenäs, Finland; 17 Institute of Oceanography, Universiti Malaysia Terengganu, Terengganu, Malaysia; 18 Maritime Affairs Unit, Joint Research Centre, European Commission, Ispra, Italy; 19 Facultad Ciencias del Mar, Universidad Católica del Norte and Centro de Estudios Avanzados en Zonas Áridas (CEAZA), Coquimbo, Chile; 20 Department of Marine Ecology, Plymouth Marine Laboratory, Plymouth, United Kingdom; 21 Educational Department, Ozeaneum Stralsund GmbH, Stralsund, Germany; 22 Department of Zoology, University of Tasmania, Hobart, Tasmania, Australia; 23 Münster, Germany; 24 Department of Biology II, Ludwig-Maximilians-University, Planegg-Martinsried, Germany; 25 National Research Institute of Fisheries and Environment of Inland Sea, Fisheries Research Agency, Hatsukaichi, Japan; University of Zurich, Switzerland

## Abstract

Species richness is the most commonly used but controversial biodiversity metric in studies on aspects of community stability such as structural composition or productivity. The apparent ambiguity of theoretical and experimental findings may in part be due to experimental shortcomings and/or heterogeneity of scales and methods in earlier studies. This has led to an urgent call for improved and more realistic experiments. In a series of experiments replicated at a global scale we translocated several hundred marine hard bottom communities to new environments simulating a rapid but moderate environmental change. Subsequently, we measured their rate of compositional change (re-structuring) which in the great majority of cases represented a compositional convergence towards local communities. Re-structuring is driven by mortality of community components (original species) and establishment of new species in the changed environmental context. The rate of this re-structuring was then related to various system properties. We show that availability of free substratum relates negatively while taxon richness relates positively to structural persistence (i.e., no or slow re-structuring). Thus, when faced with environmental change, taxon-rich communities retain their original composition longer than taxon-poor communities. The effect of taxon richness, however, interacts with another aspect of diversity, functional richness. Indeed, taxon richness relates positively to persistence in functionally depauperate communities, but not in functionally diverse communities. The interaction between taxonomic and functional diversity with regard to the behaviour of communities exposed to environmental stress may help understand some of the seemingly contrasting findings of past research.

## Introduction

While the concern about the consequences of taxon loss has spurred a burst of studies on the relation between diversity, both as driver and as response, with ecosystem functioning and compositional stability (reviewed by [1], [2]), a general agreement on the magnitude and even the direction of this role has not yet been reached [1], [3]–[5]. This is particularly true for marine ecology which is lagging behind terrestrial research on this issue [6]. Historically “biodiversity” (mostly understood as species richness) has been considered as favourable in some way or other to stability and functioning of ecosystems (reviewed by e.g. [1], [3], [7]). However, in contrast to earlier views, under ecologically realistic conditions species richness has recently been shown to relate weakly, not at all, or negatively to community stability [8], [9]. Several model approaches and a few experimental findings have postulated that species richness may even decrease stability regarding community composition [e.g. 10]. Since more than a decade the debate about the role of biodiversity at the ecosystem level is unresolved (e.g. [11]). Likely causes for the often contradictory results are that the relation between diversity and ecosystem stability or function is highly contingent on the metric of diversity used [6], [12], [13], the kind of system property investigated [5], [14], the response variables chosen [3], [5], the number of trophic levels considered [6], the spatial scale employed [15], the duration of the investigation (immediate response versus long term re-structuring) [16]–[18], the experimental concept (small synthetic assemblages versus natural communities, field versus micro- or mesocosm studies) [17], [19], and the study areas investigated [17], [19], [20]. This realization has generated pressing calls not to reduce “biodiversity” to species richness [6], [12], to scale up spatially [6], [12], [15], [21], to include multiple trophic levels [6], to allow sufficient time for population level responses [16], [17], to add observational field studies using natural communities and natural multivariate stress [2], [6], [14], to consider multivariate responses [14], and/or to clearly define stability [5]. In recent years efforts have been made to identify the common denominator for the diversity-stability relationship based on the recognition of causes for past divergent results. Much of the discussion on the discrepancies among these studies boils down to the question whether small, short, but well controlled in vitro experiments represent the real world where direct cause-effect relationships are difficult to establish because of co-varying environmental factors (e.g. [22]). Since the quality of experiments has improved and their weaknesses are increasingly recognized, some authors think that an extrapolation to natural communities is possible [18], [23], [24], while others contest this [25]–[27]. To resolve this issue, the call for more natural experiments (see above) and the request for a sound replication among ecosystems [28] or regions [23] became louder. In the investigation presented here we tried to realize the recommendations and avoid the shortcomings mentioned above. We investigated the relation between biodiversity (and unoccupied substratum) of benthic communities and their capacity to maintain their structure and composition when subjected to rapid environmental change.

A major threat to ecological communities and their diversity is rapid environmental change as caused by, for instance, habitat degradation, species invasions, or shifts in marine current regimes [29], [30]. A key question in times of rapid or gradual environmental change or fluctuations is how well a community resists or recovers from pulse stress or pressure stress with regard to either its functional or compositional properties where a compositional shift will often be accompanied by a shift in community processes (e.g. [31]). Thus, studies on the diversity – stability relationship have used as stability metric the maintenance of either function (e.g. productivity) or structure (e.g. taxonomic composition). These two community properties differ markedly from each other [5]. Ecosystem functions may respond faster to stress and return more easily to pre-stress conditions as compared to changes in taxonomic composition which react with more inertia and are less easily reversible. For the present investigation we quantified the rate of re-structuring as a response variable of marine communities to environmental change. While this may not be identical to the classical concepts of community stability (but see [5]), it is related to it by representing a quite permanent alteration of community properties and possibly entailing shifts in ecosystem services when lost species are not replaced by functionally equivalent ones. When a community structurally re-organizes under the influence of environmental change it is gradually replaced by another community composed of different species which cope better with the new conditions. This new community may be functionally equivalent or not to the original community. To avoid confusion in terminology, however, in the following we will employ the term persistence to describe the capacity to resist re-structuring under environmental change. In this sense a community is considered non-persistent (“unstable” sensu [5], [14], [16]) when an environmental shift provokes a compositional re-organization by disappearance of sensitive species and establishment of new species, driven by direct and indirect environmental impacts at the species level, by invasion events and/or by shifts in biotic interactions [10], [14], [16]. Conversely, a persistent (as employed in this paper) community withstands an environmental shift with less or slower compositional change than a non-persistent community.

System properties that have been suggested to contribute to community stability in various ways comprise unoccupied space [32], functional richness [3], [21] and - most prominently – taxonomic richness (e.g. [1], [3], [5], [33]). Since no general consensus has been found to date regarding their relative importance [34], we decided to investigate the relationship between these three system properties and the capacity of communities to persist structurally when exposed to a pressure stress which consisted in a translocation between moderately different habitats. In order to improve the generality of the results and to take into account the warnings that artificially assembled communities may not be representative of the real world (e.g. [35]), that the diversity-stability relation may be context-specific (e.g. [28]), and that the relationship may vary among ecosystems (e.g. [23]), we chose a novel approach of combining small scale, moderately controlled experiments on natural communities with large scale global replication. We test the hypothesis that compositional persistence is greater when there are less unutilised resources (as substrata for growth), higher taxonomic richness, and greater functional richness (i.e. the within-community diversity in body size, growth form, feeding mode, reproduction).

## Methods

### Ethics statement

Vertebrate animals were not part of this study. All organism handling and subsequent procedures were in accordance with European laws [European Communities Council Directive of November 24, 1986 (86/609/EEC)] and the national ethics regulations of the participating countries.

### General approach

In eight different biogeographical regions (see below), we translocated over 500 natural fouling communities among two sites within a region and assessed the relation between the rate of compositional change following the translocation and the three system properties chosen, i.e. unoccupied substratum, functional richness, and taxonomic richness.

### Experimental sites

Two suitable experimental sites were selected in each of the 8 biogeographic regions Tasman Sea (Australia), South West Atlantic (Brazil), South Pacific West (New Zealand), North West Pacific (Japan), West Pacific (Malaysia), South East Pacific (Chile), North Sea (England), and Baltic Sea (Finland) (Table 1). Within each region the 2 experimental sites were between 0.5 and 120 km apart and differed moderately in numerous abiotic and biotic features (Table 1) such as salinity, temperature, exposure, degree of pollution, kind of natural substratum as potential source for recruits, number and identity of sessile taxa or of their consumers. These between-site differences produced fouling communities (see below) differing compositionally to a variable degree. Since communities in each region had assembled on identical substratum at the same depth, for the same time and in the same season the average community dissimilarity between the two sites in each region (dissimilarity ANOSIM R, Primer Ltd) is considered a proxy of the cumulated abiotic and biotic differences between the two sites in a region and, consequently, the magnitude of the environmental change caused by the subsequent transplantation of half of the communities between the two sites (see below). This approach seems reasonable, because the composition of the pool of planktonic colonizers and consumers, as well as abiotic conditions of a given site do have a major influence on recruitment and successional dynamics (e.g. [36]–[38]). The average dissimilarity between communities from different sites in a given region ranged from 0.2 (quite similar) to 0.95 (very dissimilar) (Table 1).

**Table 1:** Site characteristics.

Country	Marine Region	Site	Coordinates	Location	Name	Temp.	Salinity	Poll.	Expos.	Source	Taxon richness^1^	Functional richness^1^	Dissim.(spec.)^2^
Australia	South Pacific, Tasman Sea	A	42°57′S, 147°21′E	marine reserve	Crayfish Point	10 to 20	marine	1	3	hb>sb	6.7(2–13)	4.6(2–8)	0.31
		B	42°54′S, 147°20′E	marina	Derwent Sailing Squadron	9 to 22	marine	3	1	mb>hb	5.9(3–10)	3.4(2–5)	
Brazil	South-West Atlantic, Rio de Janeiro State	A	23°00′S, 042°00′W	marine reserve	Arraial do Cabo	22 to 25	marine	1	1	hb	9.0(7–12)	7.0(6–9)	0.8
		B	22°52′S, 043°08′W	harbour	Mocanguê Island	23 to 27	29–35	2	1	mb>hb	12.5(9–19)	7.2(5–12)	
Chile	South-East Pacific, La Herradura Bay	A	29° 59′S, 071°22′W	bay	Univerisidad Católica del Norte	13 to 20	marine	2	2	hb	7.1(4–10)	6.9(4–9)	0.2
		B	29° 58′S, 071°21′W	bay	Compañia del Pacifico	13 to 20	marine	2	1	hb	7.4(5–12)	7.1(5–11)	
England	North-East Atlantic, North Sea	A	54° 41′N, 001°11′W	marina	Hartlepool	13 to 16	marine	3	1	mb>hb	2.7(1–8)	2.4(1–5)	0.94
		B	54°54′N, 001°21′W	marina	Sunderland	15 to 19	24–34	3	1	mb>hb	4.2(1–8)	2.8(1–5)	
Finland	North East-Atlantic, Baltic Sea, Gulf of Finland	A	59°50′N, 23°15′E	marine reserve	Ångbåtsbryggan	11 to 22	6	1	1	hb	4.5(3–6)	4.5(3–6)	0.32
		B	59°50′N, 23°16′E	marine reserve	Brännskär	11 to 21	6	1	2	hb	4.8(4–6)	4.8(4–6)	
Japan	North-West Pacific, Tokyo Bay,	A	35°66′N, 139°92′E	lagoon	Gyotoku Reserve	10 to 25	20 to 24	2	1	mb, hb	9.6(3–16)	5.7(2–7)	0.91
		B	35°67′N, 139°93′E	harbour	Ichikawa Port	10 to 25	10 to 24	3	2	mb, hb	6.9(3–16)	4.1(2–8)	
Malaysia	North-East Pacific, South China Sea	A	05°37′N, 103°04′E	estuary	Merang	30	30	2	3	sb>hb	6.7(5–9)	3.7(3–6)	0.48
		B	05°31′N, 102°57′E	coral reef	Bidong	30	31	1	3	sb, hb	6.3(4–8)	4.1(2–8)	
New Zealand	South Pacific, Hauraki Gulf	A	36°19′S, 174°47′E	harbour	Leigh Harbour	18 to 23	marine	1	1	hb	5.9(3–8)	4.9(2–7)	0.37
		B	3616′S, 174°00′E	outdoor mesocosms	Leigh Marine Laboratory	17 to 25	marine	1	2	hb, sb	5.87(2–9)	3.0(1–5)	

Temperature (Temp.) and Salinity: range during the experiment, “marine” = 35–37 psu. Pollution (Poll): 1 = unpolluted, 2 = moderately polluted, 3 = heavily polluted. Exposure (Expos): 1 = sheltered, 2 = moderately exposed, 3 = fully exposed. Source = nearby habitats where recruits presumably stem from: hb = hard bottom, including artificial hard substrata, sb = sandy bottom, mb = muddy bottom.

1 : panel averages for numbers of taxa and functional groups. Dissimilarity (Dissim, ANOSIM R) as obtained by a dissimilarity analysis comparing the taxon composition of random pairs of panels from site A and site B within a given region on the day of transplantation. The larger R, the more dissimilar are the communities regarding composition and/or relative abundance of taxa. Since paired communities in each region were identical with regard to most aspects (age, substratum, depth) their dissimilarity is considered a proxy of the cumulated abiotic (water chemistry, pollution, exposure…) and biotic (pool of recruits, consumers,…) differences between sites and, thus, the strength of the transplantation treatment.

### Fouling communities

Two to four months prior to the treatment (see below) in each site of each region 48 roughened PVC panels (15×15 cm) were suspended vertically in a water depth between 0.5 and 1 m. The experiments in different hemispheres were run during the respective spring/early summer to ensure comparability. In contrast to artificially assembled communities, the taxa within these communities co-occur and interact naturally.

### Treatment

On the day of treatment all remaining communities (variable numbers were lost at the different sites) were taken to the lab in cooler boxes. Taxon identity (to the lowest possible level) and abundance, number of functional groups, and percentage of unoccupied substratum per panel were assessed. In the following we will use the term taxon richness rather than species richness since many of the organisms could not be identified to species level for lack of appropriate identification keys regarding certain groups or in a given region. Functional richness was measured as the number of groups (containing one or more taxa) which, as adults, differ in at least one trait within four functional categories considered of ecological importance, namely body size, growth form, trophic type, and whether solitary or colonial (Table 2) [39]. By this approach, we sub-sampled the categorization scheme proposed by [40], selecting the traits of best ecological relevance in these particular communities of sessile taxa (Supplementary Table S1). Adult body size was considered important because it directly relates to space requirements and biomass production and indirectly with longevity and metabolic rates (e.g. [41]). Whether a sessile species grows upright or encrusting, in a bushy or filamentous shape affects its competitiveness by defining its need for primary substratum and its three-dimensional space of harvesting resources (light, nutrients, food). Solitary and colonial life histories differ by reproductive mode and the capacity to occupy adjacent available substratum. Finally, the mode of energy achievement is relevant for performance and competitiveness (e.g. [42], [43]). The four functional traits were subdivided into two to five levels (Table 2). In a previous study we have shown that with this resolution (yielding a total of 114 plausible functional groups) functional diversity of sessile marine communities is described as well as by an approach with a more than 10-fold higher resolution (1484 functional groups) [39]. The traits used showed variable degrees of correlation among each other. However, no test that required independence was run and even correlated system properties may convey different and complementary information about the functionality of a species. Thus, of all traits modularity and trophic type did correlate closest (r = 0.55) but it makes sense to include both traits since the first represents a mode of reproduction, a capacity of multiplying a genotype, and of pre-empting settlement substratum, while the second typifies a mode of energy acquisition.

**Table 2:** Traits used for functional grouping.

Adult body size	Growth form	Trophic type	Modularity
S<1 mm	E encrusting	A autotroph	S solitary
M 1–10 mm	M massive	P predator	C colonial
L 10–100 mm	B bushy	S suspension feeder	
XL 100–1000 mm	F filamentous	D deposit feeder	
XXL>1000 mm		G grazer	

Four ecologically relevant functional metrics were selected which are largely independent of each other but can be surrogates for other traits. Body size, for instance, correlates closely with longevity or metabolic rate. According to this scheme, a barnacle would belong to the functional group MMSS by being medium sized, of massive growth form, a suspension feeder and solitary. Larval dispersal and adult motility were not included because all taxa considered in this study did not differ in this regard having recruited from the plankton and being sessile. (For a more detailed discussion of the ecosystem service associated with these and similar traits see Bremner et al. 2006, Wahl 2009).

After this characterization of the communities, half of the communities were translocated between the two sites within a region while the other half was back-transferred to their site of origin to control for transfer effects without site change. After the transfer, the communities transplanted from site A to site B were considered “introduced” (to B) while the communities from B and back-transplanted to B were considered “resident” to B. This treatment was done in both directions, so that each site possessed a batch of introduced and resident communities. Random pairs of introduced and resident panels were suspended side by side (distance ca 10 cm) at the same place and depth where the resident communities had assembled over the past months. The response of the communities to the treatment (translocation or back-transplantation) was assessed by comparing the rates of their compositional changes during the following six to 12 weeks. The mean rate of compositional convergence within each pair of translocated and resident community over the first three weeks following translocation was expressed as the slope of the convergence curve during this time. This period was chosen, because during the phase immediately following translocation restructuring is most directly influenced by the environmental change imposed and the community properties as assessed on the day of translocation. Both influences will fade with convergence. Additionally, convergence rates (see below) are generally linear during this initial phase turning to asymptotic in a later phase. Rates of change were quantified as changes in similarity (assessed by Bray-Curtis using the software Primer® on untransformed data of species abundances per panel) to a reference community per unit time. To assess the rate of compositional change of a given community after transfer or back-transfer, its structure three weeks after the treatment was compared to its own structure on the day of transfer. The acceleration of these change rates in transferred as compared to non-transferred communities of the same origin (i.e. of the same initial composition) was used to quantify the impact of stress associated with the change of environmental conditions. The capacity of structural persistence of the transferred communities under the imposed stress was quantified by the speed of their convergence towards resident communities at the new site, i.e. the decrease of initial dissimilarity between introduced and resident communities in a given site. As (inverse) metric of persistence we preferred convergence towards resident communities over structural change as compared to original structure (i.e. self-similarity) because the former depends more directly on mortality of introduced species and invasion of resident species. Slower rates of convergence reflect a longer persistence of the original community characteristics and, thus, indicate higher resistance to the imposed environmental change. Consequently, our measure of community stability (persistence) is the inverse of the mean convergence rate within pairs of introduced and resident communities.

### Statistical analyses

Effects of various predictors on the speed of convergence between transplanted and resident fouling communities were analysed using a mixed-effects-model [44]. It was fitted with the linear mixed model formula lme (implemented in the NLME library) in the R environment (version 2.11.1) [45] and model parameters were estimated with maximum likelihood. We specified a maximal model with “Taxonomic Richness”, “Functional Richness”, the amount of available “Substratum” and all possible interactions as fixed effects. The random terms we included were considering the different levels of spatial replication in our design: “Biogeographic Region” and “Experimental Site”. Due to their hierarchical nature, we nested “Experimental Site” in “Biogeographic Region”. Stepwise model simplification then helped to identify the minimal adequate model that necessitates the lowest number of parameter estimates [46]. Graphical diagnostics in R were used to confirm normality of errors (normal-probability plots) and homogeneity of variances (fitted values vs. residuals plots). To identify the variance components for the random effects we used the linear mixed model formula lmer (implemented in the LME4 library) [47]. We used t-tests to compare the regression slopes between convergence rates and taxon richness in low and high functional richness sites, as well as the change rates of introduced and resident communities. The relation between these slopes and functional richness was tested using Spearman Rank Correlation to reduce the influence of outliers [following 48].

## Results and Discussion

The translocation represented an environmental change, the impact of which decreased over time as more and more introduced taxa were replaced by resident taxa. As is typical for all natural communities, both the introduced and the resident communities continued to change in composition after the day of translocation as a result of succession, seasonality and/or stochastic events. However, averaged over all communities, introduced communities changed faster by 29% relative to the background dynamics assessed in the resident communities of the same provenance (t-test, n = 545, t = 8.9, p<0.0001). The accelerated re-structuring was driven by two processes: (i) mortality under the new conditions; and (ii) recruitment by taxa (“invasion”) belonging to the local species pool of the target site but previously not present in the introduced community. Mortality could have been caused by intolerance towards the new abiotic conditions, lack of conspecific recruits, or sensitivity towards new biotic threats such as parasites or consumers. Circumstantial evidence suggested that predation (mostly by fishes), at least in some regions, was heavier on introduced than on resident communities, but this difference was not rigorously quantified. At all sites, the re-structuring provoked a convergence of introduced communities towards local resident communities.

Convergence rates varied among sites and regions between 0.1% and 13.5% of similarity (Bray-Curtis) increase per week. Communities in Malaysia and New Zealand changed rapidly, while those at other sites appeared more persistent (Fig. S1). The mean regional convergence rates did not relate to the biodiversity of the region (assessed as sum of all taxa found on the panels at both regional sites, r^2^ = 0.017, p = 0.76) or to the change imposed (expressed as initial dissimilarity between introduced and resident communities, Table 1, r^2^ = 0.0002, p = 0.98). In contrast, the speed of convergence related strongly to regional mean temperature (r^2^ = 0.69, p = 0.007). This could merely have reflect the well known trend of metabolic rates of poikilotherms being faster and generation times being shorter in warmer regions (e.g. [49]). This effect of temperature differences and other regional particularities injected a ‘regional noise’ into the relation between diversity and stability. To account for the regional variability, biogeographic regions and sites were included as random factors in our analysis for relationships between compositional stability and the three system properties taxon richness, functional richness and available substratum.

Most of these relationships have been studied before, but rarely simultaneously in a single experiment and never with a similar generality for different biogeographic regions (but see [50] for taxon richness – ecosystem function relationships of artificially assembled communities in three regions). The linear mixed model analysis revealed significant effects of available substratum, taxon richness and the interaction between taxon and functional richness on community persistence (Table 3, Supplementary Table S2). The strength and sometimes even the sign of these relationships varied among regions (Figs. S3, S4) demonstrating the necessity to replicate at a large scale when trying to generalize about the relative importance of taxonomic and functional richness in contributing to community persistence. Across all sites and regions convergence rate and available substratum were not related significantly (Fig. S2). At the site level, however, six out of 16 relationships between available substratum and convergence were significant (Fig. S3), four of them positive (one site each in England, Japan, New Zealand and Tasmania) and two of them negative (one site each in Chile and Japan). Available substratum can be expected to suppress structural persistence, since it is a prerequisite for the recruitment of new colonizers [51] or for dominance shifts by lateral growth of residents. The inverse relationship, accelerated convergence on panels with less available substratum, cannot be explained at present.

**Table 3:** Effects of “Taxonomic Richness”, “Functional Richness” and available “Substratum”.

*Fixed effects*	Parameter	Standard error	DF	t-value	p-value
Intercept	7.81	2.06	525	3.79	<0.001
Tax. Richness	−0.92	0.18	525	−5	<0.001
Funct. Richness	0.19	0.28	525	0.65	0.51
Substratum	0.02	0.009	525	2.17	<0.05
Tax. Richness × Funct. Richness	0.07	0.03	525	2.55	<0.05

Diversity and substratum effects on the variation in the speed of convergence between transplanted and resident fouling communities. Results from linear mixed-effects analysis. The different levels of spatial replication, i.e. “Biogeographic Region” (n = 8), and “Experimental Site” (n = 16) nested in “Biogeographic Region”, were fitted as random effects.

Species richness has been postulated to facilitate [32], [52] or hinder [51] invasions – one of the convergence drivers in the present study. Species richness may also determine the response of communities to environmental change because the susceptibilities to stress of the different species composing a community may not co-vary [53]. As a consequence, when stress sensitivity varies among species within a given functional group, the risk of stress impact at the level of ecosystem service is reduced (e.g. [54]) despite possible structural changes.

In the present study, taxon richness related negatively to convergence rate (i.e. enhanced persistence) in six of the 16 sites (two Japanese sites, and one site each in England, Finland, New Zealand and Tasmania) whereas it related positively to convergence rate in only one New Zealand site (Fig. S4). This enhancement of structural persistence by taxon richness must, however, be viewed with caution, since it interacts significantly with functional richness. Though the relevance of functional richness is attested by this interaction, its direct effect on compositional persistence, averaged across all levels of taxon richness, was not significant.

**Figure 1:**
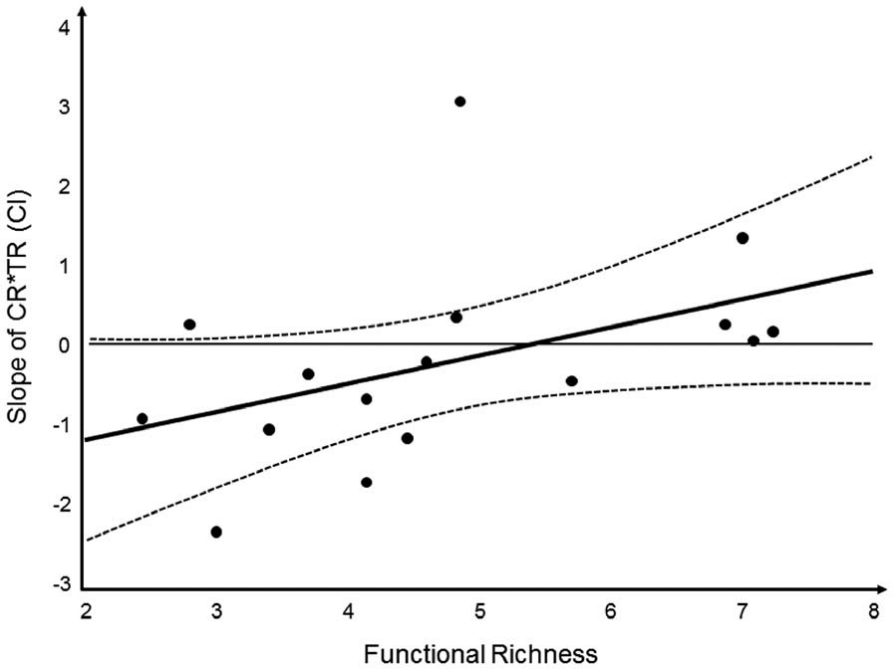
Relation between taxonomic richness and re-structuring with increasing functional richness. Average slope (±95% CI) of the relation between convergence rate (CR) and taxon richness (TR) depicted against mean functional richness. For clarity, only site means without scatter bars are shown.

**Figure 2:**
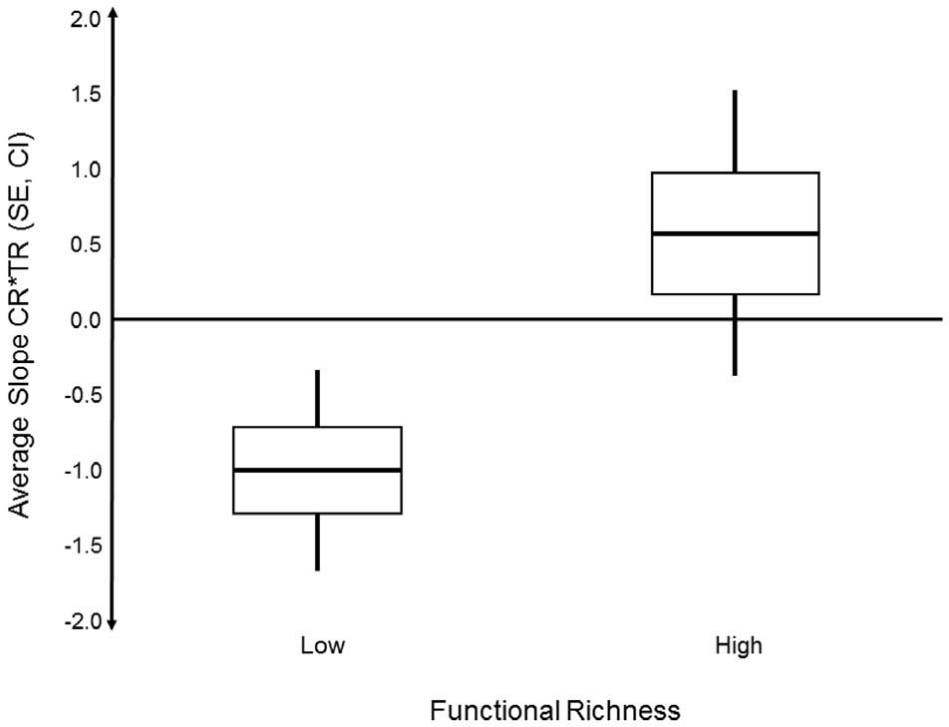
Mean relation between taxonomic richness and re-structuring at functionally poor sites and functionally rich sites. Average slopes of the relation between convergence rate (CR, box = SE, whiskers = 95% CI) and taxon richness (TR) stratified by sites with higher (”High") versus sites with lower (”Low") functional richness. The results of a pairwise t-test of the 2 samples are given.

The interaction between taxon richness and functional richness indicates that the impact of the former on structural persistence under stress (i.e. convergence rate) depends on the level of the latter. Indeed, the slope of the regression between convergence rate and taxon richness increased from negative (“enhancing persistence”) to positive (“reducing persistence”) with increasing functional richness of the experimental sites (Fig. 1, Spearman rank correlation, n = 16, r = 0.54, p = 0.03). In sites with lower functional richness (<4.5 functional groups per panel) convergence rates decreased with increasing taxon richness (significantly so as suggested by the confidence intervals in Fig. 2), while in sites with higher functional richness (≥4.5 functional groups per panel) convergence tended to accelerate with taxon richness. Thus, taxon richness enhanced community persistence under environmental change significantly more at low functional richness than at high functional richness (Fig. 2, t-test, df = 14, t = 3.2, p<0.01). Of the variance not explained by substratum or diversity effects, 53% could be attributed to the random factors “country” (illustrating the regional differences among experiments) and a further 2.2% to the random factor “sites within countries”.

At present we can only speculate about the interaction between taxon richness and functional richness regarding the compositional stress resistance of benthic communities. It should be noted that the selection of functional traits is always based on expert guessing and it cannot be excluded for our approach that a different choice might have produced a stronger (or weaker) effect of functional diversity. Meanwhile, the interaction detected suggests that the different combinations of functional richness and taxon richness encountered in this study represent different positions on the continuum between complementarity and redundancy and offer some room for interpretation. The extremes of this continuum would be i) 1 species per functional group when functional richness is high relative to species richness and ii) many species in only 1 functional group when functional richness is minimal. Under scenario (i) all species are functionally different resulting in maximum functional complementarity. Under scenario (ii) all species are functionally similar resulting in minimal functional complementarity and maximal functional redundancy. Functional complementarity is thought to enhance resistance to invasion [e.g. 55], [56] which was considered one of the drivers of convergence in our experiment. Functional complementarity is determined by the number of different functional groups present in a community and not by the number of taxa per functional group. This would explain why at elevated functional richness (high complementarity) the rate of convergence is not related to species richness (Fig. 2). Redundancy, on the other hand, has long been recognized as an insurance against the impact of species loss from a community (e.g. [57], [58]). Species loss driven by the imposed environmental change can lead to the loss of functional groups when redundancy is low (i.e. only one species per functional group), and a reduction in functional diversity enhances the risk of invasions (see above). The loss of certain functions (e.g. UV shading or chemical defense against consumers [59]) may accelerate the loss of further species. Loss of functional groups should be more severe when functional richness is already low from the start. This would explain why at low functional richness higher species richness (more redundancy) makes communities less vulnerable to environmental change. Indeed we observed that persistence of communities is strongly related to species richness at low functional richness and little related to species richness at high functional richness. The interplay between functional redundancy (reducing the consequences of species loss) and functional complementarity (reducing the risk of invasion) seem to explain the observed interactive effects of species and functional richness with regard to community level impacts of environmental change.

Our initial hypothesis was partially confirmed. Available substratum in most sites destabilized communities as expected, however, the effect of functional richness is more indirect than expected, i.e. it modulates the strength of the stabilizing effect of taxonomic richness. The variation in responses between geographic locations illustrates the complexity of how the relationships between taxonomic and functional richness help communities persist. We conclude that the drivers of compositional persistence in marine fouling communities exposed to environmental change (i.e. one aspect of stability) are multivariate and interactive. Considering only single community properties in diversity-stability studies must forcibly produce variable results in different settings.

## Supporting Information

Figure S1 **Average convergence rates per region depicted against the total taxon richness in the same region.** CR = convergence rate, SE = standard error, Aus = Australia (Tasmania), Bra = Brazil, Chi = Chile, Fi = Finland, GB = England, Jp = Japan, Mal = Malysia, NZ = New Zealand.
(TIF)

Figure S2 **Average convergence rate depicted against mean available substratum.** For clarity, only site means without scatter bars are shown. Slope and 95% confidence interval depicted.
(TIF)

Figure S3 **Re-structuring and available substratum.** Convergence rates (between paired panels) depicted against available substratum on introduced panel, stratified by region and site. Red lines indicate regression lines. Black squares indicate cases of significant (p<0.05) regressions.
(TIF)

Figure S4 **Re-structuring and richness.** Convergence rates (between paired panels) depicted against taxon richness on introduced panel, stratified by region and site. Red lines indicate regression lines. Black squares indicate cases of significant (p<0.05) regressions. Functional richness per panels is indicated as a colour gradient from blue (low) to red (high).
(TIF)

Table S1 **List of taxa and their functional traits.** In the majority of cases the “taxa” were individual species but could not be identified to a lower taxonomic level due to the lack of appropriate keys in several regions. Abbreviations of functional traits are as given in Table 1 of the article.
(DOCX)

Table S2 **Regression slopes (mean, standard error, test statistic t, significance p) between convergence rate (CR) and taxon richness (TR) and between convergence rate (CR) and available substratum (Substr) stratified by sites and regions.** n = number of pairs analysed per site.
(DOCX)
