# Supplementary figures and images for: Re-Structuring of Marine Communities Exposed to Environmental Change: A Global Study on the Interactive Effects of Species and Functional Richness
Source: PLoS One. 2011 May 18;6(5):e19514. doi: 10.1371/journal.pone.0019514 (PMC3097188; doi:10.1371/journal.pone.0019514)

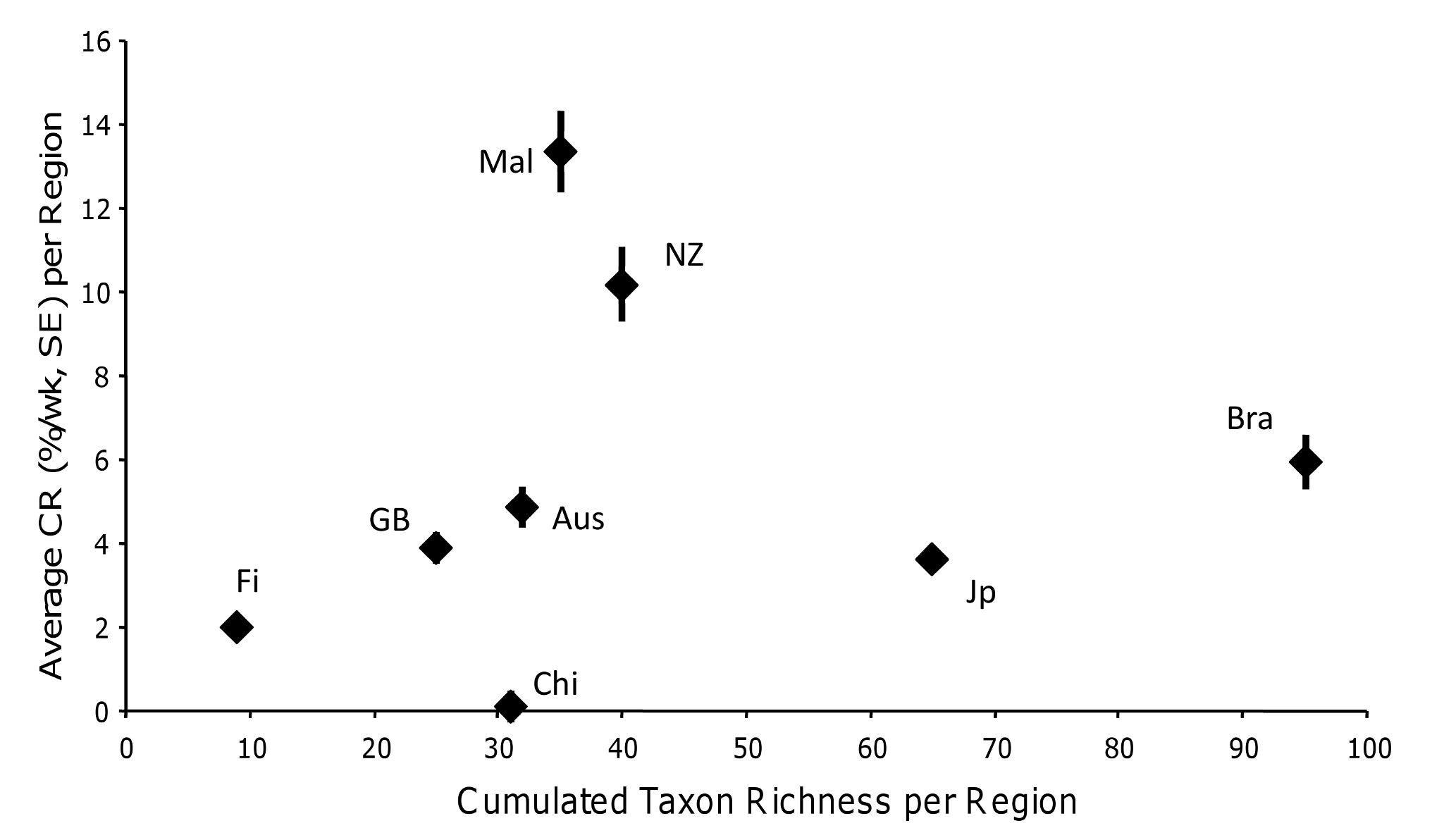

Supplement: Figure S1 — Average convergence rates per region depicted against the total taxon richness in the same region. CR = convergence rate, SE = standard error, Aus = Australia (Tasmania), Bra = Brazil, Chi = Chile, Fi = Finland, GB = England, Jp = Japan, Mal = Malysia, NZ = New Zealand. (TIF) [file pone.0019514.s001.tif]

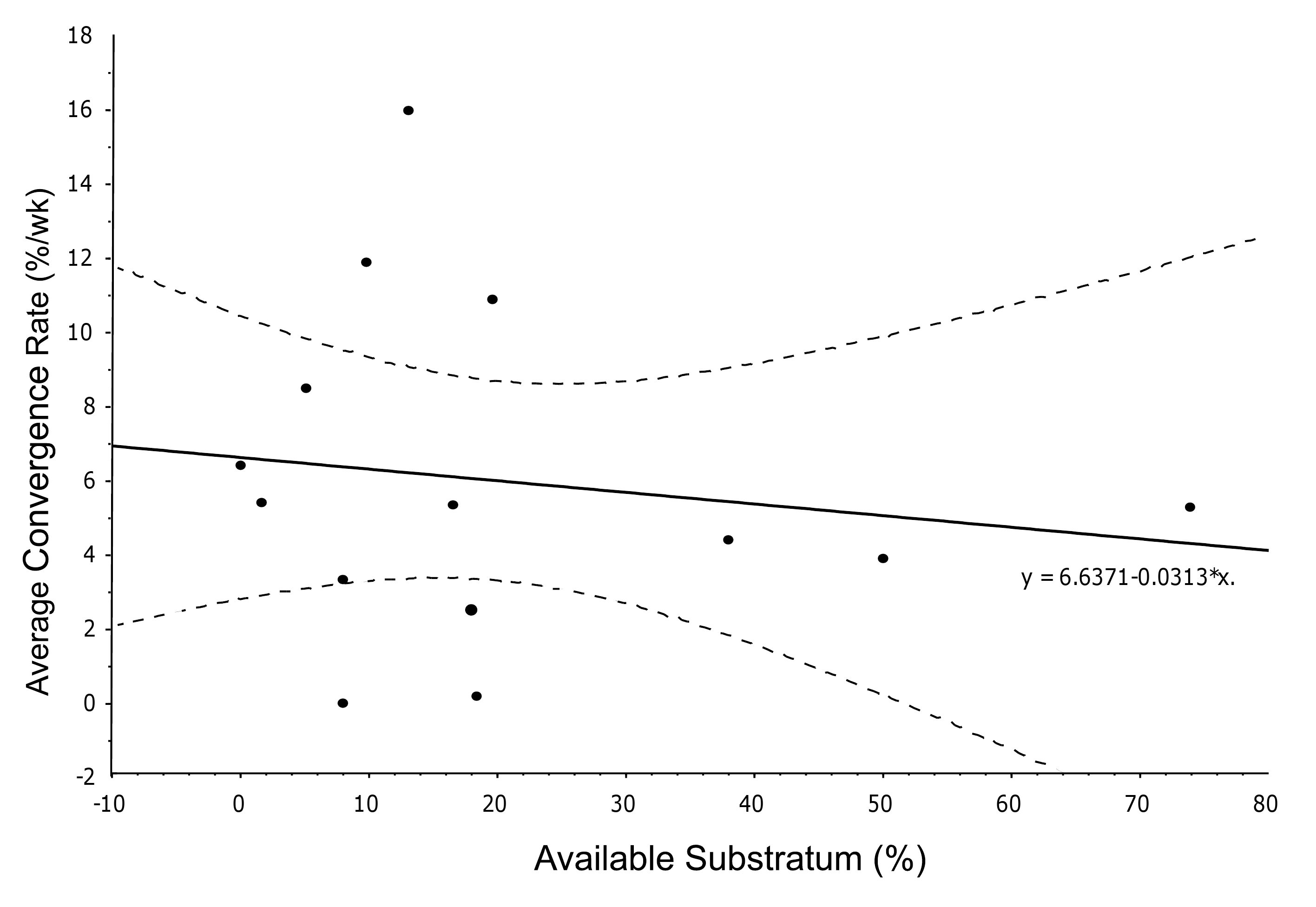

Supplement: Figure S2 — Average convergence rate depicted against mean available substratum. For clarity, only site means without scatter bars are shown. Slope and 95% confidence interval depicted. (TIF) [file pone.0019514.s002.tif]

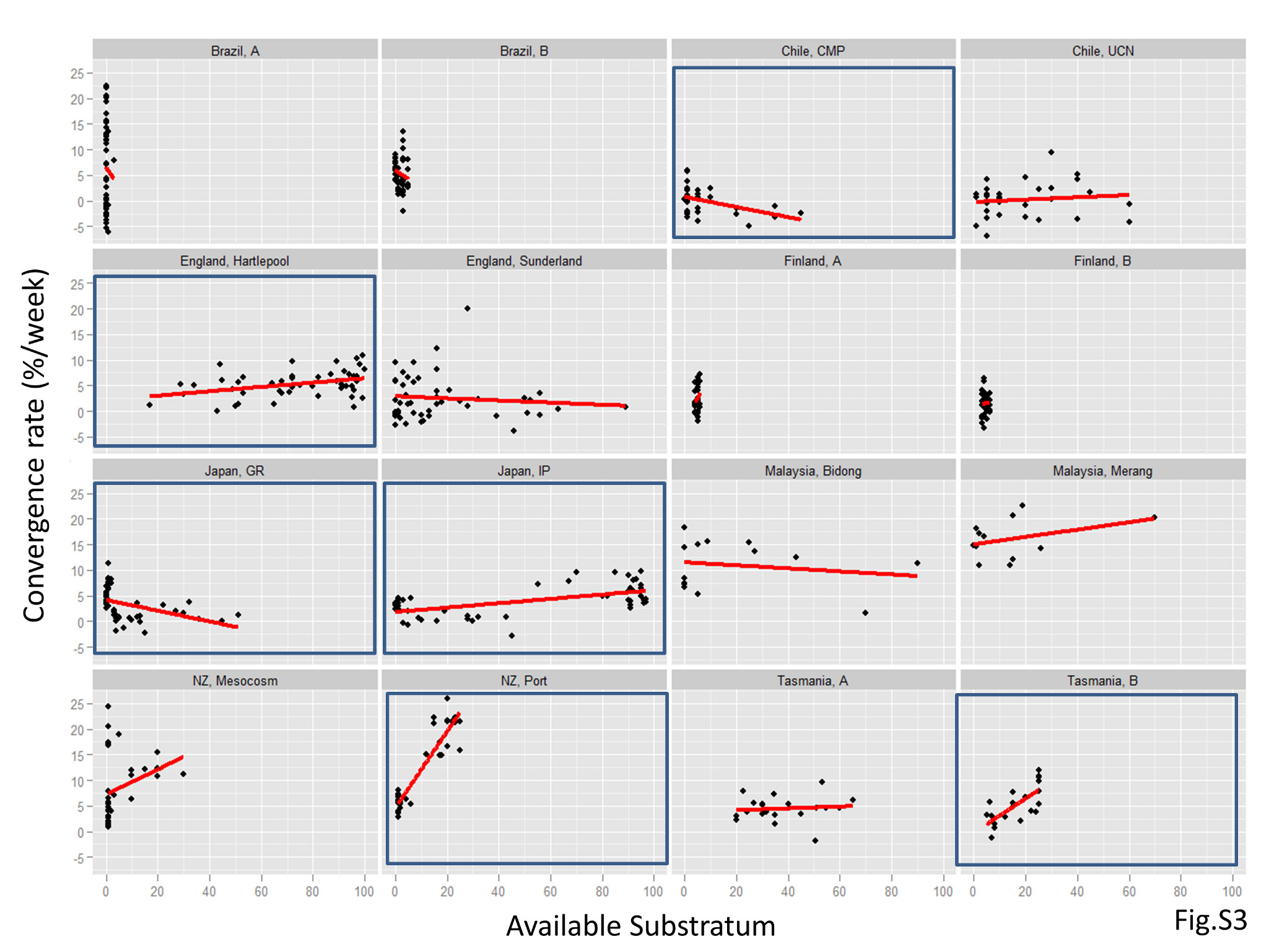

Supplement: Figure S3 — Re-structuring and available substratum. Convergence rates (between paired panels) depicted against available substratum on introduced panel, stratified by region and site. Red lines indicate regression lines. Black squares indicate cases of significant (p<0.05) regressions. (TIF) [file pone.0019514.s003.tif]

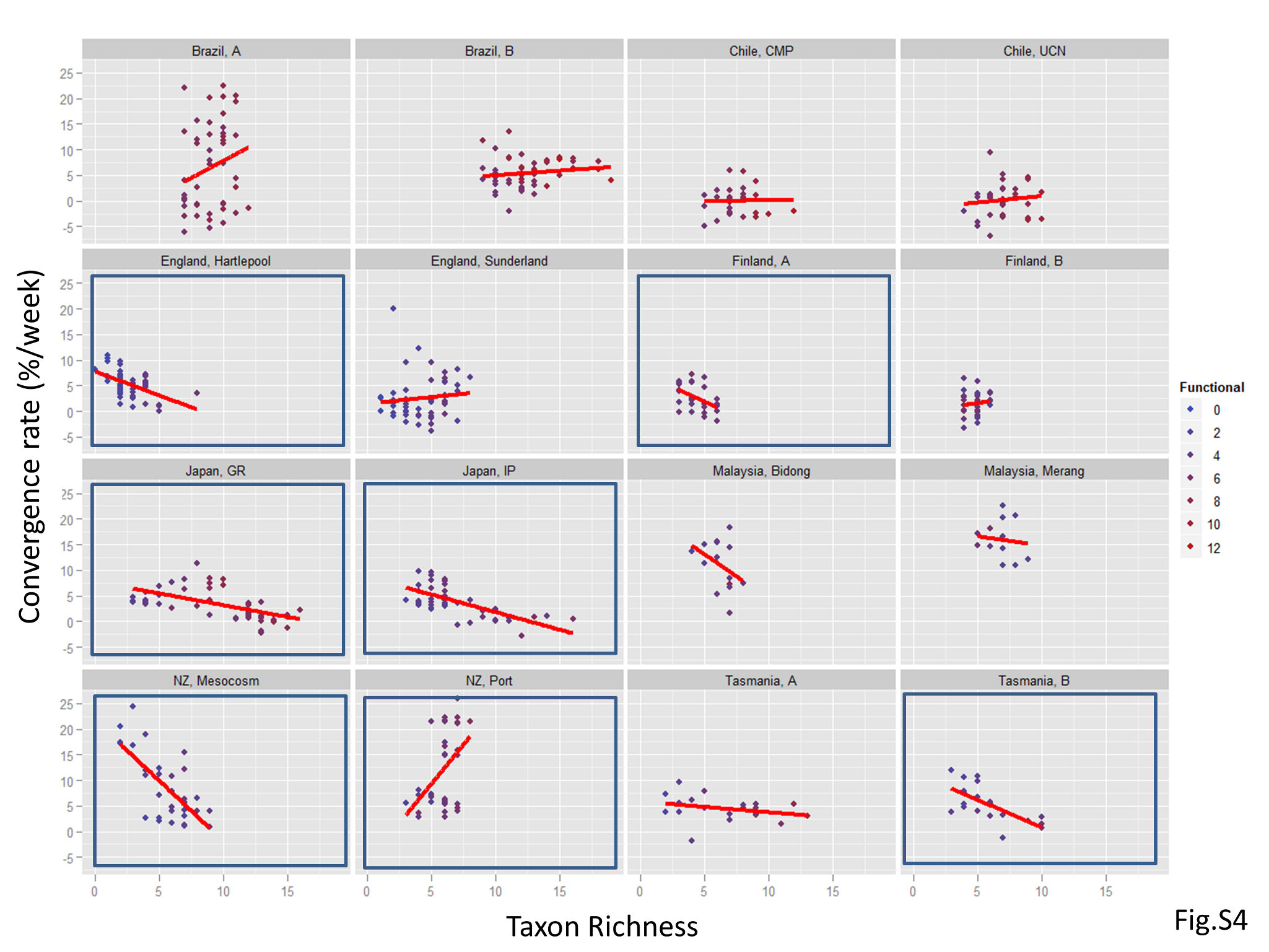

Supplement: Figure S4 — Re-structuring and richness. Convergence rates (between paired panels) depicted against taxon richness on introduced panel, stratified by region and site. Red lines indicate regression lines. Black squares indicate cases of significant (p<0.05) regressions. Functional richness per panels is indicated as a colour gradient from blue (low) to red (high). (TIF) [file pone.0019514.s004.tif]
