# Supplementary material for: Re-Structuring of Marine Communities Exposed to Environmental Change: A Global Study on the Interactive Effects of Species and Functional Richness
Source: PLoS One. 2011 May 18;6(5):e19514. doi: 10.1371/journal.pone.0019514 (PMC3097188; doi:10.1371/journal.pone.0019514)
Supplement: Table S1 — List of taxa and their functional traits. In the majority of cases the “taxa” were individual species but could not be identified to a lower taxonomic level due to the lack of appropriate keys in several regions. Abbreviations of functional traits are as given in Table 1 of the article. (DOCX) [file pone.0019514.s005.docx]

Supplementary Table S1

| **Taxon** | **Size** | **Growth Form** | **Trophic Type** | **Modularity** | **Functional group** |
| --- | --- | --- | --- | --- | --- |
| Amphipoda sp. 2 | M | M | D | S | MMDS |
| Amphipoda sp. 2 | M | M | D | S | MMDS |
| Amphipoda sp. 2 | M | M | D | S | MMDS |
| Amphipoda sp. 3 | M | M | D | S | MMDS |
| Antithamnion densum | L | B | A | S | LBAS |
| Ascidiacea sp. 15 | L | E | S | C | LESC |
| Ascidiacea sp. 21 | M | M | S | S | MMSS |
| Ascidiella aspersa | L | M | S | S | LMSS |
| Asperococcus bullosus | L | F | A | S | LFAS |
| Austromegabalanus psittacus | L | M | S | S | LMSS |
| Bacillariophyceae sp. 2 | S | E | A | S | SEAS |
| Bacillariophyceae sp. 3 | S | E | A | S | SEAS |
| Bacillariophyceae sp. 5 | S | E | A | S | SEAS |
| Bacillariophyceae sp. 7 | S | F | A | S | SFAS |
| Balanomorpha sp. 2 | L | M | S | S | LMSS |
| Balanomorpha sp. 2 | L | M | S | S | LMSS |
| Balanus albicostatus | M | M | S | S | MMSS |
| Amphibalanus amphitrite | M | M | S | S | MMSS |
| Balanus crenatus | L | M | S | S | LMSS |
| Balanus decorus | L | M | S | S | LMSS |
| Amphibalanus eburneus | L | M | S | S | LMSS |
| Amphibalanus improvisus | M | M | S | S | MMSS |
| Balanus reticulatus | L | M | S | S | LMSS |
| Balanus sp. 10 | L | M | S | S | LMSS |
| Balanus sp. 11 | L | M | S | S | LMSS |
| Balanus trigonus | L | M | S | S | LMSS |
| Botrylloides leachi | X | E | S | C | XESC |
| Botrylloides nigrum | L | E | S | C | LESC |
| Botryllus giganteum | X | E | S | C | XESC |
| Botryllus schlosseri | L | E | S | C | LESC |
| Bryopsis sp. 2 | L | B | A | S | LBAS |
| Bryopsis sp. 4 | L | B | A | S | LBAS |
| Bryozoa sp. 11 | L | M | S | C | LMSC |
| Bryozoa sp. 12 | L | B | S | C | LBSC |
| Bryozoa sp. 3 | L | B | S | C | LBSC |
| Bugula flabellata | L | B | S | C | LBSC |
| Bugula neritina | L | B | S | C | LBSC |
| Bugula sp. 1 | L | B | S | C | LBSC |
| Bugula sp. 2 | L | B | S | C | LBSC |
| Bugula stolonifera | L | B | S | C | LBSC |
| Bugula turrita | L | B | S | C | LBSC |
| Calcarea sp. 3 | L | M | S | S | LMSS |
| Calcarea sp. 4 | L | M | S | S | LMSS |
| Calothrix contarenii | M | E | A | C | MEAC |
| Caprellidae sp. 2 | M | M | P | S | MMPS |
| Caulerpa sp. 1 | X | M | A | S | XMAS |
| Centroceras clavulatum | L | B | A | S | LBAS |
| Centroceras minutum | L | B | A | S | LBAS |
| Ceramium rubrum | L | B | A | S | LBAS |
| Ceramium sp. 11 | L | B | A | S | LBAS |
| Ceramium sp. 8 | L | B | A | S | LBAS |
| Ceramium tenuicorne | L | B | A | S | LBAS |
| Chaetomorpha sp. 3 | L | F | A | S | LFAS |
| Chlorophyta sp. 2 | M | B | A | S | MBAS |
| Chlorophyta sp. 3 | L | F | A | S | LFAS |
| Chlorophyta sp. 8 | L | M | A | S | LMAS |
| Chlorophyta sp. 9 | L | F | A | S | LFAS |
| Chthamalus montagui | L | M | S | S | LMSS |
| Ciona intestinalis | L | M | S | S | LMSS |
| Cirriformia tentaculata | L | M | S | S | LMSS |
| Cladophora glomerata | X | B | A | S | XBAS |
| Cladophora rupestris | X | B | A | S | XBAS |
| Cladophora sericea | X | B | A | S | XBAS |
| Cladophora sp. 5 | L | B | A | S | LBAS |
| Cladophora sp. 7 | L | B | A | S | LBAS |
| Cladophora vagabunda | X | B | A | S | XBAS |
| Clavelina lepadiformis | L | M | S | C | LMSC |
| Codium decorticatum | X | B | A | S | XBAS |
| Codium sp. 3 | L | M | A | S | LMAS |
| Codium spongiosum | L | M | A | S | LMAS |
| Colpomenia sp. 1 | L | M | A | S | LMAS |
| Colpomenia sp. 2 | L | M | A | S | LMAS |
| Corallina sp. 5 | L | E | A | S | LEAS |
| Corallinaceae sp. 1 | L | B | A | S | LBAS |
| Cordylophora caspia | L | B | S | C | LBSC |
| Corophiidae sp. 1 | M | M | D | S | MMDS |
| Crassostrea gigas | X | M | S | S | XMSS |
| Crepidula onyx | L | M | S | S | LMSS |
| Crepidula sp. 4 | L | M | S | S | LMSS |
| Cryptosula pallasiana | L | E | S | C | LESC |
| Cyanobacteria sp. 1 | L | E | A | C | LEAC |
| Cyanobacteria sp. 2 | L | E | A | C | LEAC |
| Cyanobacteria sp. 3 | S | E | A | S | SEAS |
| Diadema sp. 1 | L | B | S | C | LBSC |
| Dictyota sp. 5 | X | B | A | S | XBAS |
| Didemnidae sp. 2 | L | E | S | C | LESC |
| Didemnidae sp. 8 | X | E | S | C | XESC |
| Didemnum speciosum | X | E | S | C | XESC |
| Didemnum vanderhorsti | X | E | S | C | XESC |
| Diplosoma listerianum | X | E | S | C | XESC |
| Diplosoma sp. 5 | X | E | S | C | XESC |
| Echinoidea sp. 2 | L | M | G | S | LMGS |
| Ectocarpus siliculosus | X | B | A | S | XBAS |
| Ectocarpus sp. 2 | X | B | A | S | XBAS |
| Ectocarpus sp. 3 | X | B | A | S | XBAS |
| Ectocarpus sp. 4 | X | B | A | S | XBAS |
| Ectopleura sp. 2 | X | F | S | C | XFSC |
| Electra crustulenta | L | E | S | C | LESC |
| Electra pilosa | L | E | S | C | LESC |
| Elminius modestus | L | M | S | S | LMSS |
| Enteromorpha intestinalis | X | F | A | S | XFAS |
| Enteromorpha sp. 3 | X | F | A | S | XFAS |
| Enteromorpha sp. 3 | X | F | A | S | XFAS |
| Enteromorpha sp. 7 | X | F | A | S | XFAS |
| Feldmannia columellaris | L | B | A | S | LBAS |
| Ficopomatus enigmatus | L | M | S | S | LMSS |
| Fistulobalanus kondakovi | L | M | S | S | LMSS |
| Galaxaura sp. 1 | L | B | A | S | LBAS |
| Galeolaria caespitosa | L | M | S | S | LMSS |
| Galeolaria hystrix | L | M | S | S | LMSS |
| Gelidiaceae sp. 1 | L | B | A | S | LBAS |
| Gracilaria sp. 1 | X | B | A | S | XBAS |
| Grandidierella sp. 1 | M | M | G | S | MMGS |
| Haliplanella lineata | L | M | S | S | LMSS |
| Haliplanella sp. 1 | L | M | S | S | LMSS |
| Herposiphonia pacifica | L | B | A | S | LBAS |
| Hiatella arctica | L | M | S | S | LMSS |
| Hiatella australis | L | M | S | S | LMSS |
| Hildenbrandia lecannellieri | L | E | A | S | LEAS |
| Hinksia sp. 1 | L | B | A | S | LBAS |
| Hydroides dianthus | L | M | S | S | LMSS |
| Hydroides sp. 1 | L | M | S | S | LMSS |
| Hydrozoa sp. 10 | L | B | S | C | LBSC |
| Hydrozoa sp. 14 | L | B | S | C | LBSC |
| Hydrozoa spp. 11 | L | B | S | C | LBSC |
| Hypnea sp. 2 | X | B | A | S | XBAS |
| Jania sp. 3 | L | B | A | S | LBAS |
| Jellyella eburnea | L | E | S | C | LESC |
| Lagenicella variabilis | L | E | S | C | LESC |
| Laomedea flexuosa | L | B | S | C | LBSC |
| Lepas anatifera | X | M | S | S | XMSS |
| Lobophora variegata | X | B | A | S | XBAS |
| Megabalanus coccopoma | L | M | S | S | LMSS |
| Megabalanus tintinnabulum | L | M | S | S | LMSS |
| Megalomma sp. 1 | L | F | S | S | LFSS |
| Melita sp. 1 | L | M | G | S | LMGS |
| Membranipora isabelleana | L | E | S | C | LESC |
| Membranipora membranacea | L | E | S | C | LESC |
| Membranipora savartii | L | E | S | C | LESC |
| Membranipora sp. 1 | L | E | S | C | LESC |
| Microcosmus exasperatus | L | M | S | S | LMSS |
| Molgula manhattensis | L | M | S | S | LMSS |
| Musculista senhousia | L | M | S | S | LMSS |
| Mycale (Carmia) microsigmatosa | L | M | S | C | LMSC |
| Mytilidae sp. 1 | L | M | S | S | LMSS |
| Mytilidae sp. 2 | L | M | S | S | LMSS |
| Mytilopsis sallei | L | M | S | S | LMSS |
| Mytilus edulis | L | M | S | S | LMSS |
| Mytilus galloprovincialis | L | M | S | S | LMSS |
| Obelia sp. 2 | X | B | S | C | XBSC |
| Obelia sp. 7 | X | B | S | C | XBSC |
| Oscillatoria laetevirens | L | E | A | C | LEAC |
| Ostrea angasi | L | M | S | S | LMSS |
| Ostrea sp. 3 | L | M | S | S | LMSS |
| Padina australis | X | B | A | S | XBAS |
| Padina sp. 2 | X | B | A | S | XBAS |
| Pectinidae sp. 1 | L | M | S | S | LMSS |
| Perna perna | L | M | S | S | LMSS |
| Perna viridis | L | M | S | S | LMSS |
| Petricola lithophaga | L | M | S | S | LMSS |
| Phaeophyta sp. 12 | L | F | A | S | LFAS |
| Phaeophyta sp. 14 | X | F | A | S | XFAS |
| Phaeophyta sp. 18 | L | M | A | S | LMAS |
| Phaeophyta sp. 6 | M | B | A | S | MBAS |
| Phaeophyta sp. 7 | M | B | A | S | MBAS |
| Phallusia nigra | L | M | S | S | LMSS |
| Planostrea pestigris | L | M | S | S | LMSS |
| Plumularia setacea | L | B | S | C | LBSC |
| Pododesmus patelliformis | L | M | S | S | LMSS |
| Polychaeta sp. 3 | L | E | S | S | LESS |
| Polydora cornuta | L | F | S | S | LFSS |
| Polydora sp. 1 | L | F | S | S | LFSS |
| Polydora sp. 2 | L | F | S | S | LFSS |
| Polysiphonia sp. 11 | L | B | A | S | LBAS |
| Polysiphonia sp. 12 | L | B | A | S | LBAS |
| Pomatoceros triqueter | L | E | S | S | LESS |
| Porifera sp. 10 | L | M | S | S | LMSS |
| Porifera sp. 11 | L | M | S | S | LMSS |
| Porifera sp. 12 | L | M | S | S | LMSS |
| Porifera sp. 13 | L | M | S | S | LMSS |
| Porifera sp. 16 | L | E | S | S | LESS |
| Porifera sp. 17 | L | M | S | S | LMSS |
| Porifera sp. 21 | L | M | S | S | LMSS |
| Porifera sp. 3 | L | E | S | S | LESS |
| Punctaria sp. 1 | X | F | A | S | XFAS |
| Pyura chilensis | L | M | S | S | LMSS |
| Pyura sp. 1 | L | M | S | S | LMSS |
| Rhodophyta sp. 11 | L | E | A | S | LEAS |
| Rhodophyta sp. 18 | L | F | A | S | LFAS |
| Rhodophyta sp. 19 | L | B | A | S | LBAS |
| Rhodophyta sp. 20 | X | E | A | S | XEAS |
| Rhodophyta sp. 28 | L | B | A | S | LBAS |
| Rhodophyta sp. 31 | L | F | A | S | LFAS |
| Rhodophyta sp. 32 | L | F | A | S | LFAS |
| Rivularia sp. 1 | L | M | A | S | LMAS |
| Sabellidae sp. 1 | L | F | S | S | LFSS |
| Sargassum sp. 2 | X | B | A | S | XBAS |
| Scapharca kagoshimensis | L | M | S | S | LMSS |
| Schizoporella errata | X | M | S | C | XMSC |
| Scytonema sp. 1 | M | F | A | S | MFAS |
| Scytosiphon lomentaria | X | F | A | S | XFAS |
| Scytosiphonaceae sp. 2 | X | F | A | S | XFAS |
| Semimytilus algosus | L | M | S | S | LMSS |
| Serpulidae sp. 3 | L | E | S | S | LESS |
| Spirorbis sp. 3 | M | E | S | S | MESS |
| Spirorbis sp. 4 | M | E | S | S | MESS |
| Spyridia sp. 1 | X | B | A | S | XBAS |
| Styela plicata | L | M | S | S | LMSS |
| Symplegma brakenhielmi | L | E | S | C | LESC |
| Symplegma rubra | L | E | S | C | LESC |
| Symplegma sp. 1 | L | E | S | C | LESC |
| Symplegma sp. 2 | L | E | S | C | LESC |
| Symplegma sp. 3 | L | E | S | C | LESC |
| Symplegma viride | L | E | S | C | LESC |
| Tanaidacea sp. 3 | M | F | S | S | MFSS |
| Tedania sp. 2 | X | M | S | S | XMSS |
| Tubularia sp. 3 | X | F | S | C | XFSC |
| Ulva lactuca | X | B | A | S | XBAS |
| Ulva pertusa | X | B | A | S | XBAS |
| Ulva sp. 10 | X | B | A | S | XBAS |
| Ulva sp. 11 | X | B | A | S | XBAS |
| Ulva sp. 12 | X | B | A | S | XBAS |
| Ulvales sp. 1 | X | B | A | S | XBAS |
| Ulvella sp. 2 | M | E | A | S | MEAS |
| Umbonula littoralis | L | E | S | C | LESC |
| Watersipora sp. 1 | L | E | S | C | LESC |
| Watersipora subtorquata | X | M | S | C | XMSC |
| Xenostrobus securis | L | M | S | S | LMSS |
| Zoantharia sp. 2 | L | M | S | S | LMSS |
| Zoantharia sp. 3 | L | M | S | S | LMSS |
| Zoantharia sp. 4 | M | M | S | S | MMSS |
| Zoantharia sp. 5 | L | M | S | S | LMSS |
| Zoantharia sp. 6 | L | M | S | S | LMSS |
