# Supplementary material for: Re-Structuring of Marine Communities Exposed to Environmental Change: A Global Study on the Interactive Effects of Species and Functional Richness
Source: PLoS One. 2011 May 18;6(5):e19514. doi: 10.1371/journal.pone.0019514 (PMC3097188; doi:10.1371/journal.pone.0019514)
Supplement: Table S2 — Regression slopes (mean, standard error, test statistic t, significance p) between convergence rate (CR) and taxon richness (TR) and between convergence rate (CR) and available substratum (Substr) stratified by sites and regions. n = number of pairs analysed per site. (DOCX) [file pone.0019514.s006.docx]

Supplementary Table S2:

| **Country** | **Site** | **n** | **Slopes of CR*TR** | **SE** | **t** | **p** | **Slopes of CR*Substr** | **SE** | **t** | **p** |
| --- | --- | --- | --- | --- | --- | --- | --- | --- | --- | --- |
| Brazil | A | 48 | 1.34 | 0.85 | 1.59 | 0.12 | -0.74 | 2.56 | -0.29 | 0.77 |
| Brazil | B | 48 | 0.17 | 0.18 | 0.97 | 0.34 | -0.33 | 0.26 | -1.3 | 0.2 |
| Chile | CMP | 32 | 0.05 | 0.32 | 0.15 | 0.88 | -0.1 | 0.04 | -2.71 | 0.01 |
| Chile | UCN | 32 | 0.25 | 0.4 | 0.63 | 0.53 | 0.02 | 0.04 | 0.63 | 0.53 |
| England | Hartlepool | 48 | -0.94 | 0.23 | -4.07 | 0 | 0.04 | 0.02 | 2.72 | 0.01 |
| England | Sunderland | 48 | 0.26 | 0.35 | 0.74 | 0.46 | -0.02 | 0.03 | -0.67 | 0.51 |
| Finland | A | 30 | -1.16 | 0.4 | -2.9 | 0.01 |  |  |  |  |
| Finland | B | 30 | 0.34 | 0.61 | 0.57 | 0.58 |  |  |  |  |
| Japan | GR | 48 | -0.46 | 0.1 | -4.67 | 0 | -0.1 | 0.03 | -3.12 | 0 |
| Japan | IP | 48 | -0.69 | 0.11 | -6.11 | 0 | 0.04 | 0.01 | 4.82 | 0 |
| Malaysia | Bidong | 14 | -1,73 | 1.21 | -1.43 | 0.18 | -0.03 | 0.05 | -0.63 | 0.54 |
| Malaysia | Merang | 13 | -0.36 | 0.88 | -0.41 | 0.69 | 0.07 | 0.05 | 1.32 | 0.21 |
| NZ | Mesocosms | 33 | -2.36 | 0.42 | -5.57 | 0 | 0.24 | 0.15 | 1.67 | 0.1 |
| NZ | Port | 33 | 3.06 | 1.05 | 2.91 | 0.01 | 0.74 | 0.05 | 13.6 | 0 |
| Tasmania | A | 20 | -0.21 | 0.16 | -1.28 | 0.22 | 0.02 | 0.04 | 0.44 | 0.67 |
| Tasmania | B | 20 | -1.07 | 0.27 | -3.95 | 0 | 0.33 | 0.08 | 4.26 | 0 |
